# Excessive intravenous crystalloid infusion after video-assisted thoracoscopic surgery lobectomy is associated with postoperative pneumonia

**DOI:** 10.1186/s13019-019-1024-6

**Published:** 2019-11-29

**Authors:** Rong Yang, Chengli Du, Jinming Xu, Linpeng Yao, Siying Zhang, Yihe Wu

**Affiliations:** 1 Department of Radiology, the First Affiliated Hospital, College of Medicine, Zhejiang University, Hangzhou, 310003 Zhejiang Province China; 2 Department of Thoracic Surgery, the First Affiliated Hospital, College of Medicine, Zhejiang University, 79 Qingchun Road, Hangzhou, 310003 Zhejiang Province China; 3 Department of Thoracic Surgery, the First Division Hospital of Xinjiang Corps, Aksu City, 843000 Xinjiang Autonomous Region China

**Keywords:** Postoperative pneumonia, Risk factors, Video-assisted thoracoscopic surgery lobectomy, Lobectomy

## Abstract

**Background:**

Video-assisted thoracoscopic surgery has been widely used in thoracic surgery worldwide. Our goal was to identify the risk factors for postoperative pneumonia in patients undergoing video-assisted thoracoscopic surgery lobectomy.

**Methods:**

A retrospective analysis of adult patients undergoing video-assisted thoracoscopic surgery lobectomy between 2016 and 05 and 2017–04 was performed. We used univariate analyses and multivariate analyses to examine risk factors for postoperative pneumonia after lobectomy.

**Results:**

The incidence of postoperative pneumonia was 19.7% (*n* = 143/727). Patients with postoperative pneumonia had a higher postoperative length of stay and total hospital care costs when compared to those without postoperative pneumonia. Multivariate analysis showed that body mass index grading ≥24.0 kg/m^2^ (vs. <24.0 kg/m^2^: odds ratio 1.904, 95% confidence interval 1.294–2.802, *P* = 0.001) and right lung lobe surgery (vs. left lung lobe surgery: odds ratio 1.836, 95% confidence interval 1.216–2.771, *P* = 0.004) were independent risk factors of postoperative pneumonia. Total intravenous crystalloid infusion grading in the postoperative 24 h ≥ 1500 mL was also identified as the risk factors (vs. 1000 to < 1500 mL: odds ratio 2.060, 95% confidence interval 1.302–3.260, *P* = 0.002).

**Conclusions:**

Major risk factors for postoperative pneumonia following video-assisted thoracoscopic surgery lobectomy are body mass index grading ≥24.0 kg/m^2^, right lung lobe surgery and total intravenous crystalloid infusion grading in the postoperative 24 h ≥ 1500 mL.

## Introduction

Lung resection is the main treatment for benign and malignant pulmonary tumours [1, 2]. Postoperative pneumonia (POP) is one of the most common complications and the main cause of death in patients undergoing lung resection [3–6]. The incidence of POP after lung surgery has been reported to range from 2.1 to 40.0% and is associated with increased mortality [7]. Several risk factors, such as age, the extent of resection, low forced-expiratory-volume-in-1-s (FEV1), advanced pathologic stage and chronic obstructive pulmonary disease for POP after lung resection have been identified [7, 8]. However, these studies investigating risk factors for POP after lung resection were based on small sample sizes, and it remains difficult to predict who will develop POP after lung resection [7–9]. Furthermore, to date, there is scarce literature research on risk factors for POP after video-assisted thoracoscopic surgery (VATS) lobectomy. With the popularity of minimally invasive thoracic surgery, identification of risk factors for POP after VATS lobectomy on a large cohort of patients is warranted. High-risk patients could be identified during the perioperative period, and targeting perioperative interventions in patients at high risk of POP may decrease POP frequency and mortality.

The objectives of this single-centre observational retrospective study were to identify risk factors for POP after VATS lobectomy and provide a reference for clinical prevention of POP.

## Materials and methods

### Study population and design

**Fig. 1:**
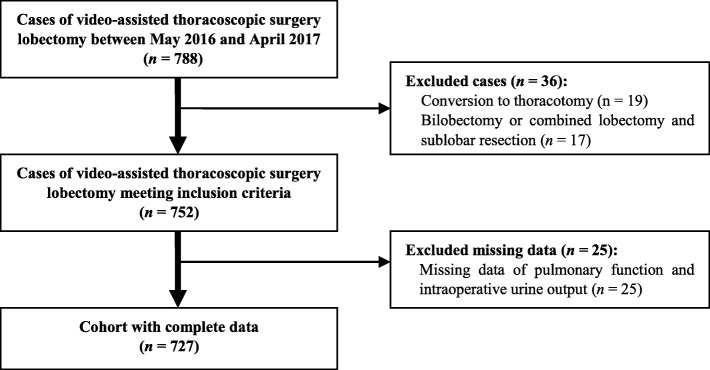
Study flow diagram illustrating surgical cohort used for data analysis

This observational retrospective study included 788 consecutive adult patients who underwent VATS lobectomy between May 2016 and April 2017 at the First Affiliated Hospital, College of Medicine, Zhejiang University (Fig. 1). Patients were considered eligible for inclusion if they were aged over 18 years and were scheduled to undergo VATS lobectomy under general anaesthesia with double-lumen intubation. Lung protective strategy of low tidal volume during single lung ventilation was managed. Excluded from the study were patients undergoing bilobectomy or combined lobectomy and sublobar resection (*n* = 17) or conversion to thoracotomy (*n* = 19). Twenty-five patients with missing data of pulmonary function and intraoperative urine output in anaesthesia records were also excluded. Finally, 727 valid cases were included (Fig. 1).

Detailed patient data of the whole cohort are shown in Table 1. Indications for surgical resection were malignant tumour (*n* = 634), benign tumour (*n* = 10), benign non-inflammatory disease (*n* = 7) and inflammatory disease (*n* = 76). Demographic, intraoperative and outcomes data were extracted from medical records, as described in Table 1. All patients received perioperative antibiotic prophylaxis. Quinolone and clindamycin were used in patients known allergy to cephalosporin. Management of postoperative pain was implemented according to a clinical practice guideline (2016) from the American Pain Society [10]. All patients started oral feedings in the sixth to eighth postoperative hour, unless they were intubated or at risk for aspiration.

**Table 1:** Perioperative factors of patients with or without postoperative pneumonia by univariate analysis (*n* = 727)

Variable	Entire cohort (*n* = 727)	Univariate analysis		
		Postoperative pneumonia (*n* = 143)	No postoperative pneumonia (*n* = 584)	*P*-value
Age (year)	62.9 ± 11.0	63.9 ± 11.8	62.6 ± 10.7	0.211
Gender (female/male)	391/336	81/62	310/274	0.444
Weight (kg)	61.6 ± 9.9	63.0 ± 10.3	61.2 ± 9.8	0.057
Body mass index (kg/m^2^)	23.2 ± 2.9	23.9 ± 2.9	23.0 ± 2.8	0.001
Body mass index grading				< 0.001
< 24.0 kg/m^2^	465 (64.0%)	71 (49.7%)	394 (67.5%)	
≥ 24.0 kg/m^2^	262 (36.0%)	72 (50.3%)	190 (32.5%)	
Smoking, pack-years	10.9 ± 23.7	8.3 ± 16.5	11.6 ± 25.1	0.477
Diabetes mellitus	64 (8.8%)	10 (7.0%)	54 (9.2%)	0.394
Hypertension	206 (28.3%)	43 (30.1%)	163 (27.9%)	0.608
Coronary heart disease	9 (1.2%)	0 (0.0%)	9 (1.5%)	0.218
History of lung surgery or chest trauma	16 (2.2%)	2 (1.4%)	14 (2.4%)	0.750
Preoperative renal insufficiency	4 (0.6%)	1 (0.7%)	3 (0.5%)	0.584
Forced expiratory volume in 1 s (percentage of predicted value)	91.1 ± 17.5%	89.9 ± 17.1%	91.4 ± 17.6%	0.364
Forced vital capacity (percentage of predicted value)	91.7 ± 15.9%	89.0 ± 14.8%	92.4 ± 16.1%	0.023
Peak expiratory flow (percentage of predicted value)	67.1 ± 23.7%	67.3 ± 27.1%	67.0 ± 22.8%	0.555
Surgical lobe				0.004
Left lung lobe	279 (38.4%)	40 (28.0%)	239 (40.9%)	
Right lung lobe	448 (61.6%)	103 (72.0%)	345 (59.1%)	
Amount of intraoperative fluids, mL				
Total	1529.2 ± 412.1	1473.4 ± 396.4	1542.8 ± 415.0	0.071
Crystalloid	1235.5 ± 385.0	1179.7 ± 354.0	1249.1 ± 391.3	0.053
Colloid	293.7 ± 255.9	293.7 ± 247.0	293.7 ± 258.3	0.999
Blood	0	0	0	–
Intraoperative bleeding, mL	48.3 ± 34.0	54.2 ± 43.3	46.8 ± 31.1	0.020
Intraoperative urine output, mL	406.7 ± 326.7	376.9 ± 315.0	414.0 ± 329.3	0.224
Length of operation, min	131.7 ± 35.6	128.2 ± 35.0	132.5 ± 35.7	0.191
Length of anaesthesia, min	159.1 ± 37.5	158.8 ± 37.6	159.2 ± 37.5	0.908
Total intravenous crystalloid infusion in the postoperative 24 h, mL	1273.5 ± 277.5	1321.2 ± 287.6	1261.8 ± 274.0	0.022
Total intravenous crystalloid infusion grading in the postoperative 24 h				0.006
< 1000 mL	128 (17.6%)	26 (18.2%)	102 (17.5%)	
1000 to < 1500 mL	461 (63.4%)	77 (53.8%)	384 (65.8%)	
≥ 1500 mL	138 (19.0%)	40 (28.0%)	98 (16.8%)	
Total intravenous colloid infusion in the postoperative 24 h, mL	0	0	0	–
Postoperative pathology				0.097
Malignant tumour *n*	634 (87.2%)	123 (86.0%)	511 (87.5%)	
Benign tumour, *n*	10 (1.4%)	0 (0.0%)	10 (1.7%)	
Benign non-inflammatory disease, *n*	7 (1.0%)	0 (0.0%)	7 (1.2%)	
Inflammatory disease, *n*	76 (10.5%)	20 (14.0%)	56 (9.6%)	
Second operation within 30 days after surgery	1 (0.1%)	0 (0.0%)	1 (0.2%)	1.000
Postoperative prolonged air leak, *n*	17 (2.3%)	6 (4.2%)	11 (1.9%)	0.120

Values are presented as mean ± standard deviation, *n*, or *n* (%)

### Variables and definitions

POP was defined as a new pulmonary infiltrate on chest X-ray (determined independently by two radiologists) with leucocytosis (leukocyte count > 10.0 × 10^9^/L) and fever (ear temperature > 38.0 °C) [11]. Only the first episode of pneumonia diagnosed during the first 7 days after surgery was studied and was defined as POP [12].

According to the Health Industry Standard of China: Adult Weight Determination (WS/T428–2013), we use body mass index (BMI) ≥24.0 kg/m^2^ as the pre-obesity standard, which is slightly different from the World Health Organization (WHO) standard (Pre-obesity: BMI ≥25.0 kg/m^2^). Preoperative renal insufficiency was defined as creatinine > 50% the upper limit of the reference range, which is 1.3 and 1.1 mg/dL for men and women, respectively [13]. The amount of total intraoperative fluids was defined as the volumes of crystalloid, colloid and blood products administered between initiation of anaesthesia care and arrival in the postanaesthesia care unit [13]. Total intravenous crystalloid/colloid infusion in the postoperative 24 h was defined as the volumes of intravenous crystalloid/colloid in the postoperative 24 h. Postoperative pathology of malignant tumour included lung cancer, atypical adenomatous hyperplasia, lymphoepithelioma-like carcinoma and lung metastasis. Inflammatory disease included bronchiectasis, tuberculosis, fungal infections, granulomatous inflammation, chronic inflammation and purulent inflammation. A prolonged air leak was defined as leak > 7 days [11]. Postoperative length of stay (PLOS) was defined as the number of hospitalised days after surgery. Hospital costs were the total hospital care costs.

### Statistical analysis

Continuous data are presented as mean ± standard deviation and were analysed using one-way variance analysis. If the variance was not homogeneous, a nonparametric test (Kruskal–Wallis H test for multiple independent samples) was used. Categorical variables are expressed as percentages and were compared by the R × C chi-square test or Fisher’s exact test, as appropriate. Binary logistic regression analyses were performed to evaluate the risk factors of POP. We created a multivariate analysis model using significant variables, as determined by the univariate analysis and suggested risk factors of POP. Additional file 1: Table S1 shows the assignment of variables in multivariate analysis. Odds ratios (ORs) were calculated from these models, together with their 95% confidence intervals (CIs). For all tests, a two-tailed *P* ≤ 0.05 was considered statistically significant. Analyses were conducted using SPSS 25.0 (SPSS, Inc., Chicago, IL, USA) [11, 13, 14].

## Results

### Patient selection and comparative univariate analysis

A total of 727 patients met our inclusion and exclusion criteria for analysis (Fig. 1). The incidence of POP was 19.7% (143 of 727). No patient died during the period of hospitalisation. The PLOS and total hospital care costs in the POP group were significantly higher than those in the no-POP group (Table 2). Table 1 summarises the perioperative factors and comparative univariate analysis results of the cohort. In univariate analysis, BMI, BMI grading, forced vital capacity (percentage of predicted value), surgical lobe, intraoperative bleeding, total intravenous crystalloid infusion in the postoperative 24 h and total intravenous crystalloid infusion grading in the postoperative 24 h were significantly different between POP and no-POP groups (Tables 1).

**Table 2:** Clinical outcomes of patients with or without postoperative pneumonia

Variable	Entire cohort (*n* = 727)	Univariate analysis		
		Postoperative pneumonia (*n* = 143)	No postoperative pneumonia (*n* = 584)	*P*-value
Postoperative pneumonia, *n*	143 (19.7%)	143	0	–
In-hospital mortality, *n*	0	0	0	–
Postoperative length of stay, d	5.9 ± 2.2	6.8 ± 3.0	5.6 ± 1.8	< 0.001
Total hospital care costs (RMB)	58,004.3 ± 9787.6	63,063.9 ± 11,011.2	56,765.4 ± 9052.4	< 0.001

Values are presented as mean ± standard deviation

### Comparative multivariate analysis of the risk factors of postoperative pneumonia

We included statistically significant factors in univariate analysis, namely, BMI grading, forced vital capacity (percentage of predicted value), surgical lobe, intraoperative bleeding, and total intravenous crystalloid infusion grading in the postoperative 24 h, in our multivariate regression model, to evaluate the preoperative predictors of POP. Binary logistics regression analysis demonstrated that BMI grading ≥24.0 kg/m^2^ (vs. <24.0 kg/m^2^: OR 1.904, 95% CI 1.294–2.802, *P* = 0.001), right lung lobe surgery (vs. left lung lobe surgery: OR 1.836, 95% CI 1.216–2.771, *P* = 0.004) and total intravenous crystalloid infusion grading in the postoperative 24 h ≥ 1500 mL (vs. 1000 to < 1500 mL: OR 2.060, 95% CI 1.302–3.260, *P* = 0.002) were independent risk factors of POP after VATS lobectomy (Table 3).

**Table 3:** Logistic model of preoperative risk factors for postoperative pneumonia in patients undergoing video-assisted thoracoscopic surgery lobectomy

Variable	OR of POP (95% CI)	*P*-value
Body mass index grading		
< 24.0 kg/m^2^	1.000	
≥ 24.0 kg/m^2^	1.904 (1.294–2.802)	0.001
Forced vital capacity (percentage of predicted value) grading		0.424
< 60%	0.931 (0.195–4.456)	0.929
60 to < 80%	1.349 (0.858–2.121)	0.195
≥ 80%	1.000	
Surgical lobe		
Left lung lobe	1.000	
Right lung lobe	1.836 (1.216–2.771)	0.004
Intraoperative bleeding grading		
< 100 mL	1.000	
≥ 100 mL	1.125 (0.659–1.919)	0.666
Total intravenous crystalloid infusion grading in the postoperative 24 h		0.008
< 1000 mL	1.163 (0.699–1.937)	0.560
1000 to < 1500 mL	1.000	
≥ 1500 mL	2.060 (1.302–3.260)	0.002

Results of binary logistics regression are presented as adjusted odds ratio (OR), 95% confidence interval (CI), and *P*-value

## Discussion

In our study, POP occurred in 143 (19.7%) of 727 patients who underwent VATS lobectomy (Table 1). Patients with POP had higher PLOS and total hospital care costs than no-POP patients (Table 2). Three independent risk factors for POP after VATS lobectomy were identified: BMI grading ≥24.0 kg/m^2^, right lung lobe surgery and total intravenous crystalloid infusion grading in the postoperative 24 h ≥ 1500 mL (Table 3).

The incidence of POP after lung resection varies. Simonsen et al. [4] reported frequencies of 3.6% and Lee et al. [9] documented a prevalence of 6.2%, whereas, Arslantas et al. [11] noted that POP occurred in 18.7% patients after lung resection. One of the reasons for this fluctuation is due to the differences in the definitions of POP. In the current study, POP were defined similarly to Arslantas et al. [11] and Allou et al. [12], including a new pulmonary infiltrate on chest X-ray, leukocyte count > 10.0 × 10^9^/L and fever. Our incidence of POP was 19.7%, which was compatible with the reported frequency [11] .

Our study showed that BMI grading ≥24.0 kg/m^2^ was an independent risk factor for POP after VATS lobectomy. The result of the present study corresponded with the earlier studies, which reported that overweight or obese patients have an increased risk of POP [15–17]. Obese patients often have reduced lung volume, altered ventilation pattern, decreased immune function, and comorbid conditions, which are risk factors for intra- and postoperative complications [16–18]. Overweight and obesity are spreading worldwide, and thoracic surgeons will encounter more overweight patients in need of surgery in the future [19]. Although a BMI ≥ 24.0 kg/m^2^ is not a surgical contraindication, it is necessary to pay close attention to overweight patients, and to strengthen respiratory exercise before lobectomy to reduce the risk of POP.

To the best of our knowledge, this is the first report to identify that right lung lobe surgery is an independent risk factor of POP after VATS lobectomy (Table 3). The incidence of POP was 23.0% (103 /448) after right lung lobe surgery and 14.3% (40/279) after left lung lobe surgery, respectively (Table 3). The reasons why right lung lobe surgery has an increasing risk of POP are unclear. The lung volume of right lung is larger than the volume of left lung (right/left lung volume, 1.22 ± 0.14) [20], so right lung lobe surgery has a greater impact on lung function and has a greater trauma than left lung lobe surgery, thus leading to an increasing risk of POP. Therefore, for patients with right lung lobe surgery, it is especially necessary to strengthen the training of respiratory function and cough ability before surgery, and take some measures to prevent lung infection after surgery, to avoid the occurrence of POP.

Finally, our study showed that total intravenous crystalloid infusion grading in the postoperative 24 h ≥ 1500 mL was an independent risk factor of POP after VATS lobectomy. In this study, POPs were all diagnosed 24 h after operation, so POP was not the reason for increased intravenous crystalloid infusion in the postoperative 24 h. Excessive intravenous fluid infusion would cause pulmonary edema and impair gas exchange, thereby placing patients at heightened risk for infection and respiratory failure [11, 13]. Shin et al. found that excessive perioperative fluid is associated with increased risk of postoperative pulmonary complications and increased 30-day mortality [13]. A meta-analysis of several trials suggested that larger fluid volumes increase the chances of postoperative pneumonia and pulmonary edema [21]. The harmful effects of fluid excess are frequently manifested in the lungs, especially after pulmonary lobectomy. Arslantas et al. conducted a study of perioperative fluid administration and the results showed that excessive perioperative infusion fluid during anatomic lung resections could increase postoperative pulmonary complications [11]. Our findings support the view that liberal postoperative fluid infusion has harmful effects on postoperative lung function and adds to the current understanding of the postoperative fluid management in several ways.

Some studies have reported risk factors for POP following lung cancer surgery. Lee noted that age ≥ 70 years, intraoperative red blood cell transfusion and forced expiratory volume in 1 s < 70% were independent risk factors of POP after lung cancer surgery [9]. Simonsen reported that major risk factors for POP following lung cancer surgery are advanced age, obesity, chronic pulmonary disease, alcoholism and atrial fibrillation [4]. The POP risk factors for VATS lobectomy in our study differ from the above studies, thus adding new content to the POP risk factors study.

Our study is one of the few to show risk factors for POP after VATS lobectomy. However, this study has potential limitations. First, it was a single-centre retrospective study. Second, antibiotics were used prophylactically in every patient, thereby masking the discovery of risk factors for POP. Finally, the study population only included adult patients who underwent VATS lobectomy, which limits the generalisability of the findings.

## Conclusions

This present study suggested that patients with POP had higher PLOS and total hospital care costs than no-POP patients. The major risk factors for POP following VATS lobectomy included body mass index grading ≥24.0 kg/m^2^, right lung lobe surgery and total intravenous crystalloid infusion grading in the postoperative 24 h ≥ 1500 mL. Clinicians should remain vigilant in preventing and treating pneumonia and other infections in patients with these risk factors. The next step for future studies is the creation of a clinical scoring system to predict POP.

## Supplementary information

**Additional file 1: Table S1.** Assignment of variables in multivariate analysis.

## Data Availability

All data generated or analyzed during this study are included in this published article and its additional file.

## Abbreviations

BMI: Body mass index
CIs: Confidence intervals
FEV1: Low forced-expiratory-volume-in-1-s
ORs: Odds ratios
PLOS: Postoperative length of stay
POP: Postoperative pneumonia
VATS: Video-assisted thoracoscopic surgery
WHO: World Health Organization
