# Supplementary material for: Excessive intravenous crystalloid infusion after video-assisted thoracoscopic surgery lobectomy is associated with postoperative pneumonia
Source: J Cardiothorac Surg. 2019 Nov 29;14:209. doi: 10.1186/s13019-019-1024-6 (PMC6884861; doi:10.1186/s13019-019-1024-6)
Supplement: Supplementary file 1 — Additional file 1: Table S1. Assignment of variables in multivariate analysis. [file 13019_2019_1024_MOESM1_ESM.doc]

**Table 1S. Assignment of variables in multivariate analysis**

| **Variable** | **Assignment instruction** |
| --- | --- |
| Body mass index grading | <24.0 kg/m^2^ = 1;  ≥24.0 kg/m^2^ = 2 |
| Forced vital capacity (percentage of predicted value) grading | <60% = 1;  60% to <80% = 2;  ≥80% = 3 |
| Surgical lobe | Left lung lobe = 1;  Right lung lobe = 2 |
| Intraoperative bleeding grading | <100 mL = 1;  ≥100 mL = 2 |
| Total intravenous crystalloid infusion grading in the postoperative 24 h | <1000 mL = 1;  1000 to < 1500 mL = 2;  ≥1500 mL = 3 |
